# It’s All About That Case: Production and Reading Fluency

**DOI:** 10.1027/1618-3169/a000615

**Published:** 2024-09-24

**Authors:** Kathleen L. Hourihan, Jonathan M. Fawcett

**Affiliations:** ^1^ Department of Psychology, Memorial University of Newfoundland, Canada

**Keywords:** production effect, recognition, font, recollection

## Abstract

**Abstract:** Prior evidence has indicated that the act of producing a word aloud is more effortful than reading a word silently, and this effort is related to the subsequent memory advantage for produced words. In the current study, we further examined the contributions of reading effort to the overall production effect by making silent reading more effortful. To do this, participants studied words that were presented in standard lowercase font format and words that were presented in an aLtErNaTiNg CaSe font format (which should be more effortful to read). Half of the words in each font condition were read aloud, and half were read silently. Participants completed an old/new recognition test. Experiment 1 was conducted online; Experiment 2 was conducted in-lab and recorded reading times at study to confirm that alternating case font slows reading. In both experiments, we found a production effect in recognition that was uninfluenced by font type. We also found that alternating case font selectively increased recollection (but not familiarity) relative to lowercase font. Thus, the additional time to read words in a disfluent font does not appear to interact with memory benefit of producing words aloud.


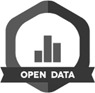


For skilled readers, reading is a relatively effortless task and is an important aspect of everyday life. Fortunately, one of the easiest ways to improve memory is to simply read things aloud instead of reading them silently. This is now known as the *production effect* (MacLeod et al., 2010). The production effect has been primarily examined in the context of studies using single words as stimuli, but it has also been shown to benefit memory for line drawings (Fawcett et al., 2012), photos (Hourihan & Churchill, 2020; Whitridge, Clark, et al., 2023), and longer text-based material (Ozubko, Hourihan, and MacLeod, 2012), but not for face–name pairs (Hourihan & Smith, 2016). Most studies on the production effect use old/new recognition testing, but the effect is observed in two-alternative forced-choice recognition (MacLeod et al., 2010; Experiment 3), fill-in-the-blank content questions (Ozubko et al., 2012), free recall (e.g., MacLeod et al., 2022), and also associative recognition (with some caveats; Putnam et al., 2014). The original explanation for the production benefit in memory, which is also still the predominant explanation, is that the act of production adds distinctive information to the encoding episode, and this information is later useful at the time of retrieval for differentiating studied from new stimuli (e.g., MacLeod et al., 2010; Ozubko & MacLeod, 2010).

However, alternative accounts have proposed that production at encoding strengthens the memory trace (e.g., Bodner & Taikh, 2012; Bodner et al., 2014), rather than necessarily adding distinctive information, and it is this strengthening that leads to the subsequent memory difference. Support for the strength account primarily comes from between-subjects or pure list manipulations of production (see Fawcett, 2013; Fawcett et al., 2023), with the idea being that distinctive information associated with reading aloud is unlikely to be informative at the time of test if all study items had been read aloud (see also Ozubko & MacLeod, 2010). Thus, the underlying cause of the production effect is indeed still up for debate, although some recent computational modeling research suggests that both distinctiveness and strength may play a role (e.g., Caplan & Guitard, 2024; Cyr et al., 2022; Jamieson et al., 2016), even in pure lists, depending on how strength is defined. The goal of the current study is to explore whether the mixed-list production effect in recognition may be caused, at least in part, by the fact that the act of production is simply more effortful than reading silently, and it is this effort that imparts the subsequent memory benefit.

One early criticism of the production effect (based on criticisms of the generation effect; e.g., Begg & Snider, 1987) was that the “benefit” in recognition observed may not have been an actual benefit in memory for produced words, but a cost in memory for the silent words. Specifically, the idea was that the requirement to produce an overt response on only some trials led participants to adopt a *lazy reading* strategy on silent trials. This idea can at least partially explain the cost/benefit trade-off that is often seen when comparing pure- to mixed-list studies in the production effect, where (for example) the hit rate for aloud words in mixed lists is comparatively higher than the total hit rate for a pure list of aloud words, while the hit rate for silent words in mixed lists is comparatively lower than the total hit rate for a pure list of silent words (e.g., Bodner et al., 2014). When participants are asked to read some of their study items aloud, this may lead to reduced processing of the silent words on that mixed list, relative to what participants would otherwise be doing if they were studying a pure list silently. However, a production effect is still observed even when all words have been generated or processed elaboratively (Forrin et al., 2014; MacLeod et al., 2010).

There is evidence that the effort involved in performing the productive act, or even just preparing to produce a word, may indeed be more effortful than reading silently. Forrin et al. (2019) showed that encoding in a mixed-list production task can be influenced by performance anticipation, reducing memory for silent items. More recently, Willoughby (2020) demonstrated that production invokes greater cognitive effort than silent reading (as measured via pupillometric changes; Hess & Polt, 1964) and that variation in the effort (as measured by the relative change in pupil dilation) afforded to production is itself predictive of the magnitude of the behavioral production effect. Thus, there is evidence that preparing to and actually producing a word aloud is more cognitively demanding than reading a word silently, with at least some evidence this effort is linked to performance benefits.

Overall, various types of item-specific distinctive information have been proposed to be added to the encoding trace by the act of production (e.g., Forrin & MacLeod, 2018; Forrin et al., 2012), including the act of planning to execute the overt reading response (e.g., Forrin et al., 2019). The overt response required on aloud trials also increases the required effort at encoding (Willoughby, 2020). Thus, the mixed-list recognition benefit associated with the act of reading a word aloud may be caused by multiple components, including the plan to produce aloud (e.g., Willoughby, 2020); the motoric operations associated with vocalizing (e.g., Forrin et al., 2012; MacLeod et al., 2010); the auditory information associated with hearing one’s voice aloud (e.g., Forrin & MacLeod, 2018); the encoding of item-specific, distinctive orthographic and phonological aspects of the word (MacLeod et al., 2010); and general cognitive effort associated with planning and executing the overt reading response.

A recent computational model of how production influences item recognition (Caplan & Guitard, 2024) addresses how some of these different components contribute to the production effect. The model considers orthographic, phonological, and semantic features as distinct sets (with provision for consideration of an action-related feature set). While semantic (deep) features are represented sparsely (i.e., with less competition among features), phonological and orthographic features are considered to be shallow and more likely to be similar to one another. Importantly, Caplan and Guitard’s model implements attentional subsetting at encoding and recognition, which influences the relative focus on particular classes of features. The act of production focuses attention on phonological features (due to the need to read the word aloud) and therefore increases the likelihood of phonological features being encoded, relative to silent reading. The overall increase in the number of encoded features operates as a strengthening of the memory trace (by increasing the length of the vector representing an item’s features) and also increases the relative distinctiveness of that item through the item-specific features that are encoded. At recognition, participants are thought to focus on phonological features (consistent with the distinctiveness heuristic as described by MacLeod et al., 2010; see also Dodson & Schacter, 2002) when considering whether a probe is studied or new; produced items have more stored phonological features and are thus more likely to lead to a match in memory than are silently read items.

In Caplan and Guitard’s (2024) model, produced items have more stored phonological features than silent items, and other feature classes (e.g., orthographic) are presumed to be equivalent between the encoding conditions. As attention is limited in capacity, let us assume that production uses all possible attentional resources to store as many features as are maximally possible to encode, and silent items therefore have fewer-than-maximal features encoded. Now, let us consider a stimulus manipulation that disrupts reading fluency, slowing down reading and drawing attention to orthographic features. For items read aloud, there must be an attentional trade-off, borrowing resources from encoding the phonological features to focus on and encode orthographic features, with overall no net change to the total number of features encoded. For items read silently, there is potential for the focus on orthographic features to result in a net increase in the overall numbers of features encoded, thus increasing the likelihood of subsequent recognition for those items. Overall, this would result in an underadditive interaction between production and font, where recognition of aloud words in a disfluent font may be similar to recognition of aloud words in a lowercase font, but silent words studied in disfluent font would show an increase in recognition, potentially equivalent to recognition of the aloud words. To examine this possibility, we compared standard lowercase font words to words presented in alternating case font, in which every other letter within a word alternates between uppercase and lowercase, which disrupts processing fluency (Rhodes & Castel, 2008; Whittlesea & Leboe, 2000).

Alternating case font was first used by Whittlesea and Leboe (2000) to disrupt the contributions of orthographic regularity to reading fluency. This manipulation was subsequently used by Rhodes and Castel (2008) in their examination of the font size illusion. This metamemory illusion is the finding that participants predict larger font words will be recalled better than smaller font words, but subsequent recall performance does not (often) show any difference based on font size at encoding (but see, e.g., Maxwell et al., 2022). Over a series of experiments aimed to dispel this illusion in their participants, Rhodes and Castel could only convince their participants to not consider font size in their memory predictions when the fluency of word reading was disrupted by presenting words in alternating case. They argued that words in large font are read more fluently, which leads participants to estimate greater recall; disrupting fluent reading eliminated the cue that participants erroneously used to predict higher recall (others have since shown that beliefs about font size effects in memory also contribute to this illusion quite substantially; e.g., Mueller et al., 2013).

The current study consisted of two experiments. The study phase was similar in both experiments: Participants were asked to learn a list of words for an upcoming memory test and were cued to read half of the words aloud at study. Memory was tested with old/new recognition. The first experiment was conducted fully online during the COVID-19 pandemic. Experiment 2 was conducted in person and served as a more controlled replication of the first experiment. In addition, vocal response times during encoding were collected in Experiment 2, as a measure of whether alternating case font was indeed more difficult to read, which should be evident in slower reading response times (see Whittlesea & Leboe, 2000). The two experiments also differed in terms of whether the originally studied font condition was reinstated at test (Experiment 1) or changed for all studied items at test (Experiment 2). To the extent that alternating case font does make reading more effortful, and this reading effort leads to a trade-off with the phonological features normally responsible for the production benefit, then the production effect magnitude should be reduced, or potentially eliminated, for alternating case font words (relative to standard lowercase font words).

## Experiment 1

Experiment 1 was conducted online in Winter 2021. (This was during the COVID-19 pandemic, when no in-person data collection was taking place. Due to limitations in online experimentation resources at the time, the experiment was conducted using Qualtrics (Qulatrics, Provo, UT) survey software). This allowed for random ordering of study trials and test trials for each participant, but the same words were always in the same conditions for all participants. Participants studied a list of words that were presented in either lowercase or alternating case font and were asked to read half of each of the words in the two font types aloud and the other half silently. Then, they completed an old/new recognition test with all studied items tested in their same font type (along with new items in the two font types). We expected to observe a production effect in recognition. It was unclear whether or how font type would influence recognition. Studies in the literature that have used alternating case font have primarily used free recall testing (e.g., Mueller et al., 2013; Rhodes & Castel, 2008), with inconsistent outcomes. In the original work of Rhodes and Castel (2008), they found no effect of font type on recall, but Mueller et al. (2013) found lower recall for alternating case font words, relative to standard lowercase font (see also Jemstedt et al., 2018; Xie et al., 2018). However, it is unclear whether recognition memory would be affected by font type in the same manner as recall.

A few studies have examined the influence of a different disfluent font manipulation, Sans Forgetica, on recognition memory (e.g., Cui & Liu, 2022; Geller et al., 2020) and found no difference in recognition performance, relative to standard font (Arial, Times New Roman). Geller and Peterson (2021) did find a recognition benefit for Sans Forgetica over Arial font, but only in participants who did not expect the upcoming recognition test. They additionally showed that participants in self-paced study conditions spent longer studying words presented in Sans Forgetica than they did studying words presented in Arial, with a corresponding increase in cued-recall performance. Speculatively, we can suggest that the disfluent processing associated with alternating case font has potential to operate as a desirable difficulty, improving recognition accuracy, but the additional time required to read the disfluent words may be primarily concentrated on the perceptual, rather than semantic level, and therefore may not influence recognition substantially (cf. Wetzler et al., 2021). Importantly, we predicted an interaction between production and font type, such that the magnitude of the production effect should be smaller for the alternating case font words than for the lowercase font words to the extent that the extra effort required to read the disfluent font case trades off with the extra effort required to produce words. That is, as described above, the alternating case font may shift attention to focus on orthography, reducing the contribution of phonology to the recognition of produced words.

### Method

#### Participants

Participants consisted of 34 undergraduate students at Memorial University of Newfoundland. They received course credit in exchange for participating. Sample sizes in this and the following experiment were determined based on the number of participants we were capable of recruiting during the academic year, with data collection ceasing once the academic year ended; in either case, the resultant sample sizes were typical of studies in this area.

#### Materials and Design

The materials consisted of a list of 160 words generated using the MRC Psycholinguistic Database (Coltheart, 1981). The study was conducted using the online survey software Qualtrics (Qualtrics, Provo, UT). Due to design limitations with the use of Qualtrics, the same words were always in the same condition for all participants, but both study and test trials were presented in a new random order for each participant. See the Appendix for details on the word lists. The current study employed a 2 × 2 within-subjects design. The first independent variable was font type; half of the words were presented in lowercase format (e.g., pressure), and the other half were presented in alternating case format (e.g., PrEsSuRe). The second independent variable was production, with half of the words read aloud (presented in blue) and the other half read silently (presented in red). The dependent variables were hits and false alarms on the old/new recognition test.

#### Procedure

Participants completed a consent form before accessing the experiment. Upon completing the consent form, participants were taken to the experiment itself. Prior to the study phase, participants were instructed that they would be studying a list of words for a later memory test. They were asked to read words presented in blue aloud and to read words presented in red silently. Participants were shown a series of 80 words, presented one a time on a plain white background screen for 2 s each. The list consisted of both words presented in a lowercase format and in an alternating case format, each group containing 40 words with 20 being read aloud and 20 being read silently.

After the study phase, participants engaged in an old/new recognition task. In this old/new recognition task, participants were shown a series of 160 words, presented one a time on a plain white background screen, and were instructed to decide whether the item was previously studied (“old”) or if it was not previously studied (“new”). They provided their response by clicking one of two radio buttons (labeled Old and New) with their computer mouse and pressing an arrow key to submit the response and move on to the next trial. The survey prevented participants from skipping trials without selecting one of the response options. Eighty of the words were previously studied, and 80 were new items (40 lowercase and 40 alternating case). Items were tested in the same format in which they were studied (either lowercase or alternating case); all items were presented in dark gray font at test. In addition, there were an equal number of new items in the two font formats.

Immediately following the old/new recognition task, participants were asked to indicate which words they were supposed to read aloud in the study and which words they actually read aloud. These questions were asked to ensure that participants correctly followed instructions in the online study.

### Results and Discussion

Of the 34 participants, four reported that they did not follow the instructions of the study. Instead of reading only the blue words aloud, they reported reading either the red words aloud or both the red and blue words. Separate analyses were conducted both with and without these four participants. Note that Bayes factors reported for each effect within a given ANOVA reflect inclusion Bayes factors (calculated using the *BayesFactor* and *bayestestR* packages in R; Makowski et al., 2019; Morey & Rouder, 2024), representing evidence favoring the inclusion of that variable in the model as compared to a comparable model excluding that factor. Also, throughout Bayes factors are reported using the convention that *BF*_10_ refers to evidence support the presence of an effect and *BF*_01_ refers to evidence against an effect; default priors were used in all cases and matching (for the inclusion Bayes factors) was set to true, reflecting the additive benefit of an interaction over a purely additive model. Bayes factors greater than 3 were considered to reflect strong evidence. η_*g*_^2^ refers to generalized eta squared (Bakeman, 2005). We primarily include Bayesian analyses as supplements to the traditional frequentist analyses, particularly to bolster the ability to interpret null or marginal effects; we include Bayes factors for all analyses reported below for completeness.

In the full sample, the effects of font type and encoding condition on recognition hits were analyzed in a 2 (font type: alternating case vs. lowercase) × 2 (production: aloud vs. silent) repeated-measures analysis of variance (ANOVA). The mean hit rates are displayed in Table 1. There was a significant main effect of font type, *F*(1, 33) = 27.24, *MSE* = 0.025, *p* < .001, η_*g*_^2^ = .131, *BF*_10_ = 5.07 × 10^5^, with recognition performance being better for alternating case font than lowercase font. Also, there was a significant main effect of production, *F*(1, 33) = 20.88, *MSE* = 0.021, *p* < .001, η_*g*_^2^ = .087, *BF*_10_ = 3.24 × 10^3^, with recognition performance being better for words that were read aloud than words that were read silently. There was no significant interaction, *F*(1, 33) = 1.25, *MSE* = 0.010, *p* = .271, η_*g*_^2^ = .003, *BF*_01_ = 3.09. False alarms to lowercase words (*M* = 0.31, *SE* = .02) were not significantly different from false alarms to alternating case font words (*M* = 0.30, *SE* = 0.03), *t*(33) = 0.147, *p* = .884, *d* = 0.025, *BF*_01_ = 5.39. Analysis of the group excluding those failing to follow instructions was qualitatively identical, with the exception that the interaction was both nonsignificant and demonstrated slightly more convincing evidence favoring the *Null*, *F*(1, 29) = 0.18, *MSE* = 0.009 *p* = .672, η_*g*_^2^ < .001, *BF*_01_ = 3.74.^1^

**Table 1:** Mean performance (with standard errors in parentheses) for font types and production conditions in Experiments 1 and 2

Measure	Lowercase font		Alternating case font	
	Silent	Aloud	Silent	Aloud
Experiment 1				
Recognition hits	.52 (0.03)	.61 (0.04)	.64 (0.03)	.77 (0.03)
Experiment 2				
Reading time	757 (24.5)	732 (20.1)	769 (25.7)	761 (23.6)
Recognition hits	.52 (0.04)	.79 (0.02)	.51 (0.04)	.81 (0.02)
Recollection (“R”)	.16 (0.03)	.40 (0.03)	.19 (0.03)	.45 (0.04)
Familiarity (“F”)	.36 (0.03)	.39 (0.02)	.32 (0.02)	.36 (0.03)
Adjusted familiarity	.44 (0.04)	.66 (0.03)	.41 (0.03)	.66 (0.04)
*Note*. Reading time on silent trials represents the time to produce a repeated “check” response aloud, rather than reading the actual word. Times are in milliseconds. Adjusted familiarity refers to the proportion of “F” responses on trials other than those on which an “R” response was provided.				

As expected, a significant production effect was observed. Although it was not necessarily predicted a priori, the overall recognition benefit for words presented in alternating case font is not entirely surprising. Alternating case font has been used previously to disrupt the fluency of processing, which should therefore increase overall encoding time and make encoding more effortful. Although previous studies have found either no differences between lowercase and alternating case in terms of memory performance (Rhodes & Castel, 2008), or a reduction in recall performance for alternating case words (Jemstedt et al., 2018; Mueller et al., 2013), these studies used free recall, and the current study used old/new recognition, which reinstated the original font context. It may be the case that the less fluent encoding of alternating case font words only offers a small boost to memory, such that it can impart a benefit on old/new recognition but is not sufficient to boost performance in the more self-guided context of recall.

Regardless, the critical prediction had been that there would be a reduced benefit of production when encoding was made more effortful, shifting attention from phonology to orthography for words read aloud. This was not observed; the production effect was of similar magnitudes for both lowercase and alternating case fonts. Thus, it may be that the recognition benefit imparted by production at encoding is not due to the effort required to produce words aloud and instead is entirely driven by the presence of additional perceptual features added to the encoding episode (hearing yourself, moving your mouth, etc.; see Forrin & MacLeod, 2018; Forrin et al., 2012). Alternatively, we had suggested above that the act of production may use full attention at encoding, such that a disfluent font that required more effort to read would necessitate borrowing attention from encoding phonological features to instead encoded orthographic features. As we did not observe the predicted interaction, production of lowercase font words may therefore not require maximal effort, and thus, the more effortful disfluent font may have encouraged encoding of more orthographic features without a concurrent cost to encoding phonological features. This resulted in the additive effects of production and font that we observed. Moreover, the recognition test re-presented items in their originally studied font, and thus, the inclusion of alternating case font words during test may have contributed to an attentional set that considered both phonology and orthography when assessing probes as old/new. However, there are a number of methodological limitations with the current study that would make such a conclusion premature.^2^ Thus, Experiment 2 was conducted in person to replicate the results of Experiment 1, but with implementing a greater degree of experimental control.

## Experiment 2

In Experiment 2, we further examined whether part of the recognition benefit of production is caused by additional effort at the time of encoding. The results of Experiment 1 suggested that alternating case font words are later recognized better than standard lowercase font words, potentially due to more time and/or effort required at encoding simply to read the word, and boosting encoding of item-specific orthographic features. However, production did not interact with this font effect. The first goal of Experiment 2 was to confirm that alternating case words are indeed more difficult to read than are lowercase font words; to do this, we recorded vocal response times to read words aloud. As a comparison, participants were asked to respond “check” aloud on all silent word trials; repeating the same response has been shown to not impart a memory benefit compared to providing no overt response at all (MacLeod et al., 2010, Experiment 4). Assuming that alternating case font does result in more effortful reading, response times to read alternating case words aloud should be slower than to read lowercase font words (and it is possible that a response time difference may be apparent with check responses on silent trials as well). The first known use of alternating case font was by Whittlesea and Leboe (2000; Experiments 9 and 10), in the context of classifying pronounceable nonwords as category members or nonmembers. Their classification task required participants to first produce the probe item aloud, and they recorded vocal response times; these showed significantly slower times to produce nonwords in alternating case than in uniform case.

Second, Experiment 2 examined whether the recognition benefit for alternating case font words observed in Experiment 1 relies on the reinstatement of the studied font context at test. That is, production effect experiments most frequently use font color at study to instruct participants whether each word should be read aloud or silently; at recognition, a third font color is used for all test items to ensure that the color context does not overlap with either of the studied conditions (but see, e.g., Fawcett et al., 2012). Thus, the production effect does not rely on reinstatement of the original studied color context (see also Bodner et al., 2020). Design limitations in Experiment 1 meant that all studied items were tested in the same font condition; it is possible that the recognition benefit observed for words studied (and tested) in alternating case font is contextually dependent, such that failing to reinstate the font at test may reduce or eliminate the recognition benefit observed. That is, inclusion of alternating case font words during test may lead participants to focus on both orthography and phonology when considering probe items; changing the font type at test may lead participants to focus primarily on phonology again (see Caplan & Guitard, 2024), reducing the influence of orthographic features previously encoded. In Experiment 2, therefore, all items were tested in a third font type: all capital letters. The production effect is predicted to remain even when the font type changes between study and test. However, it is unclear whether the recognition benefit we observed in Experiment 1 for words originally studied in alternating case font was driven primarily by encoding or retrieval. That is, it may be that the benefit relies on reinstatement of the studied font type at retrieval, such that failing to reinstate this font context may eliminate the hit rate advantage we observed for alternating case font in Experiment 1.

Third, Experiment 2 examined whether any increased effort associated with reading alternating case font words at encoding may influence the quality of memory, even if overall recognition hit rates do not differ. To this end, we incorporated recollect–familiarity judgments (akin to remember–know judgments; for a review, see Yonelinas, 2002) into the recognition portion of our task: Here, familiarity refers to a *feeling* that a given item had been studied, whereas recollection is thought to reflect a conscious re-experiencing of the encoding episode in which that item had been encountered. Production in mixed lists has been shown to enhance both recollection and familiarity, with the former tentatively attributed to distinctive encoding processes and the latter attributed to differences in attention or engagement with the items (Fawcett & Ozubko, 2016; Ozubko et al., 2012). These effects are thought to be dissociable, as the production effect is driven by familiarity alone when manipulated between-subjects – resulting in the between-subject production effect being smaller in magnitude (Fawcett, 2013; Fawcett & Ozubko, 2016; Fawcett et al., 2023; but see Whitridge, Huff, et al., 2024). In the present case, we predict a production effect for either measure.

### Method

#### Participants

Participants were 43 undergraduate students at Memorial University of Newfoundland who received course credit for participation. None had participated in Experiment 1.

#### Materials and Design

Materials consisted of a list of 160 words that were generated using the eLexicon database (Balota et al., 2007). This selection of words accounted for factors such as word length, frequency, and phonology. Word length ranged from 4 to 7 letters (*M* = 5.79, *SD* = 1.03). All words were either 1 or 2 syllables in length. The frequency had a minimum of 28 and a maximum of 414,103 (*M* = 19,314, *SD* = 49,981). Unlike in Experiment 1, words from the pool were randomly assigned to condition for each participant in the current experiment. The background screen was black for all phases of the experiment. During the practice and study phases, words to be read aloud were presented in blue font (half in lowercase and half in alternating case) and words to be read silently were presented in white font (half in lowercase and half in alternating case). All instructions were presented in yellow font; all test words were presented in purple font and in all capital letters (e.g., PRESSURE).

The current study was conducted in person, and stimuli were presented and responses recorded using E-Prime 3.0 software (Psychology Software Tools, Pittsburgh, PA) and the E-Prime response box with voice key. All participants were tested on the same desktop computer running Windows 10 Enterprise. Prior to the beginning of the practice phase, the microphone was adjusted to a height appropriate for the participant, and the keyboard was positioned on a separate desk where the experimenter used the keyboard to code response time validity (i.e., to code any voice key mis-fires or failures to appropriately detect initial voice onset). At the end of the study phase, the microphone was removed from the desk, and participants were given the keyboard to complete the test trials.

The independent variables were the same as in Experiment 1. In addition to considering hits and false alarms, the study also collected recollect versus familiar judgments at recognition. Vocal reading response latencies were also recorded during the study phase.

#### Procedure

Upon completing an informed consent form, participants were instructed that they would be studying a list of words for a later memory test. They were informed that they would be asked to read any words printed in blue aloud and to say check for any words printed in white. Prior to the study phase, participants completed a series of eight practice trials, consisting of two trials in each of the study conditions (aloud lowercase, aloud alternating, silent lowercase, silent alternating). Practice trials were presented in random order. These trials allowed the experimenter to calibrate the microphone distance and to ensure that participants were complying with the color instructions appropriately. On each trial, a fixation cross was displayed at the center of the screen for 500 ms, immediately followed by the word. When the voice key detected the onset of speech on a given trial, the word was removed from the screen, the response time was recorded (relative to word onset), and a yellow line was displayed. At this point in the trial, the experimenter pressed a key on the keyboard to code the trial as valid (the word offset at the start of the participant’s vocalization) or invalid (e.g., the voice key was not correctly triggered by the onset of the participant’s speech; the participant read the word incorrectly or read the word instead of saying check on a silent trial, etc.); this keypress also advanced to the next study trial. If no vocal response was detected after 2,000 ms had elapsed, the trial advanced to the yellow line, and the trial was coded as an error trial by the experimenter. The practice trials could be repeated as many times as needed to ensure the microphone was appropriately responding to voice onset and that participants were reading blue words aloud but saying check in response to white words.

In the study phase, participants were shown the list of 80 words, presented one at a time on a black background, for up to 2 s each. The list consisted of 40 words presented in a lowercase format and 40 in alternating case format, with 20 words in each font format presented in blue (to be read aloud) and 20 presented in white (to be read silently, with “check” said aloud). After the study phase, participants were provided with detailed instructions for the test phase. They were asked to read definitions of recollection and familiarity, and to verbally summarize the difference to the experimenter (who corrected them if their response was incorrect). Participants were instructed to press the R key if they could re-experience studying a word, the F key if it was familiar but they did not recollect it, or the N key if neither was true. The recognition test presented all 160 words from the pool in random order. The keypress response labels were presented on the screen during test trials, and words remained on the screen until the participant pressed one of the three possible response keys.

### Results and Discussion

#### Reading Response Times (RTs)

As described above, the researcher coded each study trial as valid or invalid; invalid trials include equipment misfire or participants producing an incorrect response (i.e., reading the word on a silent trial or incorrectly reading the word on an aloud trial). One participant was excluded from the response time and study phase accuracy analyses entirely (but still retained in the recognition analysis below) due to a high number of invalid microphone trials (75% of trials were invalid). This participant was retained for our analysis of recognition memory because most of the excluded trials were misfires caused by task irrelevant sounds (e.g., inhaling deeply prior to speaking) or microphone malfunctions (e.g., the participant speaking too softly to register). The average proportion of trials removed from each condition (based on the remaining 42 participants) was as follows: aloud lowercase, *M* = 0.15, *SE* = 0.03; aloud alternating, *M* = 0.11, *SE* = 0.03; silent lowercase, *M* = 0.08, *SE* = 0.02; and silent alternating, *M* = 0.09, *SE* = 0.02. These proportions were analyzed in 2 (font type: alternating vs. lowercase) × 2 (production: aloud vs. silent) repeated-measures ANOVA; the only significant effect was that there were more invalid trials in the aloud condition than in the silent condition, *F*(1,41) = 8.49, *MSE* = .011, *p* = .005, η_*g*_^2^ = .039, *BF*_10_ = 40.58. This likely reflects the fact that participants made a repeated verbal response on silent trials (i.e., “check”) that was more likely to consistently be detected by the voice key hardware, whereas the specific words read in the aloud condition varied in terms of initial phoneme, which could lead to greater variability in the likelihood of the microphone not correctly detecting the initial vocal onset, and the RA coding it as invalid. There was also a marginally significant Font Type × Production interaction, *F*(1,41) = 3.60, *MSE* = .007, *p* = .065, η_*g*_^2^ = .012, *BF*_10_ = 1.15, such that the difference in errors between aloud and silent trials was slightly larger for lowercase than alternating case fonts, but the effect was quite small (in addition to being nonsignificant) so we opted not to overinterpret it. Note that invalid trials were only excluded from the reading RT analysis, and those words (and the one participant) were retained in the recognition test analysis.

The mean valid reading trial RTs (shown in Table 1, based on the 42 participants described above) were analyzed in a 2 (font type: alternating vs. lowercase) × 2 (production: aloud vs. silent) repeated-measures ANOVA. There was a significant main effect of font type, *F*(1, 41) = 10.83, *MSE* = 1,641, *p* = .002, η_*g*_^2^ = .004, *BF*_10_ = 2.83, with slower reading times for alternating case font than lowercase font. The main effect of production was not significant, *F*(1, 41) = 2.44, *MSE* = 5,034, *p* = .126, η_*g*_^2^ = .003, *BF*_10_ = 1.23. There was no significant interaction, *F*(1, 41) = 1.78, *MSE* = 1728, *p* = .190, η_*g*_^2^ = .001, *BF*_01_ = 2.87. Analyses of log-transformed reaction times produce similar findings and qualitative conclusions.

These results serve primarily as a manipulation check of the idea that alternating case font is less fluently processed than typical lowercase font and therefore should slow down the overall time required to read a word. Our RT analysis is consistent with this idea, as both aloud and silent trials (on which participants read the word silently but said “check” aloud on each trial) were slower for alternating font than for lowercase font. Thus, these data provide evidence supporting the idea that alternating case font disrupts processing fluency and slows processing time. We next examine whether this slower processing time reduced the magnitude of the subsequent production benefit in recognition (i.e., by improving encoding of the silently read words via more effortful reading that increases encoding of orthographic features).

#### Recognition Performance

##### Old/New Accuracy

First, “R” and “F” responses were combined into a single old response to analyze hit rates. The mean hit rates are displayed in Table 1. The false alarm rate was *M* = 0.20, *SE* = .02. Hits were analyzed in a 2 (font type: alternating vs. lowercase) × 2 (production: aloud vs. silent) repeated-measures ANOVA. There was a production effect, *F*(1, 42) = 104.14, *MSE* = 0.034, *p* < .001, η_*g*_^2^ = .348, *BF*_10_ = 3.24 × 10^24^. However, the main effect of font type was not significant, *F*(1, 42) = 0.25, *MSE* = 0.011, *p* = .619, η_*g*_^2^ < .001, *BF*_01_ = 5.65, nor was the interaction, *F*(1, 42) = 1.50, *MSE* = 0.008, *p* = .228, η_*g*_^2^ = .001, *BF*_01_ = 3.22. Thus, the overall hit rates do not replicate the pattern observed in Experiment 1, in which (in addition to observing a production effect) alternating case font words were recognized better than lowercase font words. However, it is important to note that the font context was changed at test for all items in Experiment 2, whereas Experiment 1 reinstated the original font context at test. Thus, although alternating case font may disrupt encoding fluency, it may be a relatively weak cue at retrieval, such that it must be reinstated to have a positive impact on overall item recognition.^3^

To evaluate the apparent differences between our experiments empirically, and to maximize statistical power for the critical interaction, we also combined our experiments together and conducted an exploratory analysis using a 2 (font type: alternating vs. lowercase) × 2 (production: aloud vs. silent) × 2 (experiment: Experiment 1 vs. Experiment 2) mixed ANOVA. Unsurprisingly, there was a main effect of production, *F*(1, 75) = 120.60, *MSE* = 0.028, *p* < .001, η_*g*_^2^ = .232, *BF*_10_ = 2.77 × 10^25^. We also observed a main effect of font, *F*(1, 75) = 20.02, *MSE* = 0.017, *p* < .001, η_*g*_^2^ = .030, *BF*_10_ = 470.82, such that memory was better overall for the alternating case font. The main effect of experiment failed to reach significance, *F*(1, 75) = 0.42, *MSE* = 0.095, *p* = .517, η_*g*_^2^ = .004, *BF*_01_ = 3.52. Importantly, both Experiment × Font, *F*(1, 75) = 19.58, *MSE* = 0.017, *p* < .001, η_*g*_^2^ = .029, *BF*_10_ = 754.37, and Experiment × Production, *F*(1, 75) = 20.33, *MSE* = 0.028, *p* < .001, η_*g*_^2^ = .049, *BF*_10_ = 2.01 × 10^5^, interactions were significant, such that the effect of font was larger in the initial experiment, but the effect of production was larger in the second experiment. Neither the critical Font × Production, *F*(1, 75) = 2.74, *MSE* = 0.009, *p* < .101, η_*g*_^2^ = .002, *BF*_01_ = 3.18, nor the Experiment × Font × Production, *F*(1, 75) < 0.01, *MSE* = 0.009, *p* < .940, η_*g*_^2^ < .001, *BF*_01_ = 3.97, interactions were significant, and in fact, there was strong evidence against either in the aggregate sample.

##### Recollection and Familiarity

Mean estimates of recollection and familiarity are displayed in Table 1. For estimating recollection, the proportion of total test trials that had received an “R” response was used. To estimate familiarity, the proportion of the remaining test trials (i.e., total trials minus the trials on which an “R” response was provided) that had received an “F” response was used (see Fawcett & Ozubko, 2016; Ozubko et al., 2012). These two measures were analyzed in separate 2 (font type: alternating vs. lowercase) × 2 (production: aloud vs. silent) repeated-measures ANOVAs. For recollection, there was a production effect, *F*(1, 42) = 97.47, *MSE* = 0.023, *p* < .001, η_*g*_^2^ = .281, *BF*_10_ = 1.17 × 10^22^. The main effect of font type was also significant, *F*(1, 42) = 6.72, *MSE* = 0.010, *p* = .013, η_*g*_^2^ = .010, *BF*_10_ = 1.21, with greater recollection reported for items that had been studied in alternating case font compared to lowercase font. The interaction was not significant, *F*(1, 42) = 0.27, *MSE* = 0.009, *p* = .605, η_*g*_^2^ < .001, *BF*_01_ = 4.26. For familiarity, there was again a production effect, *F*(1, 42) = 63.95, *MSE* = 0.038, *p* < .001, η_*g*_^2^ = .221, *BF*_10_ = 1.64 × 10^13^. However, the main effect of font type was not significant, *F*(1, 42) = 0.43, *MSE* = 0.020, *p* = .516, η_*g*_^2^ = .001, *BF*_01_ = 5.20 , and neither was the interaction, *F*(1, 42) = 0.44, *MSE* = 0.025, *p* = .510, η_*g*_^2^ = .001, *BF*_01_ = 3.70.

Thus, although overall hit rates showed no influence of font type (and, indeed, evidence against such an effect), analysis of recollection showed that participants were more likely to report re-experiencing the study item at recognition if that word had been studied in alternating case font than when it had been studied in lowercase font. There was no effect of font on familiarity. The disruption in reading fluency at encoding associated with alternating case font therefore has no overall influence on probability of retrieval when the font is not reinstated at test but is more likely to result in a memory trace that will later be recollected. This was equally true for words read aloud and words read silently. That is, contrary to our original predictions, the magnitude of the production effect was not influenced by font type at all, despite clear evidence that words in alternating case font are read more slowly and are more likely to be recollected, relative to lowercase font words. Alternating case font therefore may be influencing subsequent memory by adding additional distinctive (orthographic) information at the time of encoding, thus influencing recollection rather than familiarity.

In addition to our conclusions related to alternating case, we also replicated earlier findings that the production effect improves both recollection and familiarity in mixed-list designs (Fawcett & Ozubko, 2016; Ozubko et al., 2012). However, we are unable to adjudicate as to the mechanisms underlying the effect for recollection or familiarity, except that insofar as the impact of alternating case derived from distinctive encoding or attentional processes, it might have been expected to interact with the relevant production effect should those processes similarly drive the benefits of production.

## General Discussion

In the current study, we examined whether the effort involved with the act of production is a significant contributor to the subsequent recognition benefit. To do this, we increased the effort required to read a word, even silently, by presenting words in alternating case font. To the extent that reading effort is even partly responsible for the production benefit (e.g., Willoughby, 2020), then when reading is made more effortful there should be proportionately less benefit of reading aloud. That is, the more effortful reading of silent words should result in an incremental boost at encoding by requiring additional attention to encode orthographic features; for words read aloud, the effort required to read disfluent font may shift attention away from phonological features in favor of orthographic features, thus reducing the remaining benefit to aloud words caused by distinctive features.

Our results did not support our original predictions. In neither experiment did we find any evidence that the magnitude of the production effect was influenced by differences in reading effort associated with the font. We did observe significant production effects in both experiments and further discovered that alternating case font itself can influence recognition memory. In Experiment 1, in which the studied font type was reinstated at test, we found increased hit rates for alternating case font words relative to lowercase font words, and no statistical difference in false alarms based on font type. The font effect did not significantly interact with the production effect, either, and we even observed evidence *against* such an effect. In Experiment 2, in which the font type was not reinstated, we did not find an effect of font on overall hit rates. However, we did find that alternating case font selectively increased recollection. Once again, we found evidence against an interaction between production and our font manipulation.

We shall first consider the effects of the font manipulation in our experiments. Although our primary goal in using alternating case font was to examine how disrupted fluency at encoding would influence the production effect, we observed some interesting effects on recognition due to the font type itself. Previous studies have used alternating case font with the goal of disrupting reading fluency (Whittlesea & Leboe, 2000), primarily in examinations of how fluency contributes to metamnemonic predictions (Rhodes & Castel, 2008; see also Jemstedt et al., 2018; Mueller et al., 2013). Each of these previous studies used free recall testing, finding either no effect of font type on recall (Rhodes & Castel, 2008) or lower recall of words studied in alternating case font (Jemstedt et al., 2018; Mueller et al., 2013; see also Xie et al., 2018). However, as the primary focus of these studies was on memory predictions, not recall per se, there was relatively little discussion of how the font was influencing actual encoding.

In our study, we used old/new recognition testing, which has different demands on the participant at retrieval than does recall. In Experiment 1, we reinstated the original font context and found that alternating case words were more likely to be recognized than lowercase font words. Importantly, the hit rate increase was not accompanied by a concordant increase in false alarms, which suggests a boost to actual memory sensitivity, rather than a difference in response bias based on font. This was supported by our results in Experiment 2; although no overall hit rate differences were observed, words studied in alternating case font were more likely to be subsequently recollected than words studied in lowercase font. In this experiment, all words were tested in a third font format of all capital letters. Although this font format did not overlap with words originally studied in lowercase font, it did have partial overlap with the words originally studied in alternating case font (i.e., because half of the letters in each word were indeed studied in capitals).^4^ However, as we did not observe an effect of font in overall hit rates in this experiment, it suggests that the partial reinstatement of studied font was not sufficient to influence overall probability of recognition but may have potentially contributed to the observed boost in recollection. Although it is not certain whether this would replicate without partial reinstatement of studied font, we did observe that the subjective experience of remembering having studied a given word is more detailed and recollective when words were first encountered in alternating case font. When that font is reinstated at recognition, this context reinstatement appears to be sufficiently powerful to increase the likelihood of identifying an item as old (i.e., potentially influencing familiarity in this case). Based on the Caplan and Guitard (2024) model, we can speculate that inclusion of the two study phase font types at test in Experiment 1 encouraged participants to focus attention on both phonology and orthography when assessing probe words, whereas testing items in a changed font context in Experiment 2 led participants to focus primarily on phonology.

Interestingly, alternating case font may be a special case in terms of its positive influence on recognition memory. Yue et al. (2013) examined whether blurring text at the time of study may operate as a desirable difficulty based on reasoning similar to that applied to alternating case font (Rhodes & Castel, 2008): Blurred text should be less fluent to process. However, they found that blurred text resulted in reduced recall relative to clear text, and no difference in recognition (Yue et al., 2013; Experiment 2b). Other studies examining disfluent fonts (Sans Forgetica) found reduced recall for the unusual font, despite the font being designed to reduce forgetting (Maxwell et al., 2022), and Sans Forgetica has not been shown to have any consistent influence on recognition memory (Cui & Liu, 2022; Geller et al., 2020), other than when participants are not expecting a test (Geller & Peterson, 2021). Thus, there is opportunity for further study of whether alternating case font can be applied as a desirable difficulty at encoding under varying conditions.

Turning to the production effect itself, our results contribute to the larger literature showing the robustness of the production effect in recognition; we found large magnitude production effects in both experiments. Our original motivation in using the disfluent font at study was to increase the effort required to read words silently, and thus, we predicted that the added benefit of reading aloud should be reduced to the extent that effort is an important contributor to the overall production effect. This prediction was based on past findings that reading aloud and preparing to do so are cognitively effortful (Forrin et al., 2014; Willoughby, 2020). However, contrary to our predictions, the production effect was not reduced for alternating case font words. Thus, although there is evidence that reading aloud is indeed more effortful than reading silently (and we showed that reading alternating case words is more effortful than reading lowercase words), it does not appear as though reading aloud requires maximal use of attentional resources, and thus, additional effort at encoding adds to the resultant production benefit in recognition memory, rather than reducing it (but see Willoughby, 2020). This finding is broadly aligned with past studies showing that generation likewise fails to interact with the production effect (Bodner et al., 2020; Forrin et al., 2014; MacLeod et al., 2010).

Consequently, we return to the distinctiveness account to explain the mixed-list production effect in recognition memory (MacLeod et al., 2010; Ozubko & MacLeod, 2010). That is, when words are read aloud at encoding, additional distinctive information associated with the act of production is encoded along with the target item, and that additional information can help increase the likelihood of subsequent recognition. Caplan and Guitard (2024) model this as primarily including additional phonological information for words that are produced. In the context of our studies, it may be that the alternating case font is an additional orthographic detail that is likely to be encoded as part of the original trace. However, encoding of the font context does not appear to be different when the word is read aloud versus silently (see also Bodner et al., 2020), such that more effortful reading is additive with production benefits.

Despite the fact that Experiment 2 replicated the finding that production improves memory as measured by either recollection or familiarity in mixed-list designs (e.g., Ozubko et al., 2012), neither interacted with alternating case. Although the genesis of the production effect in either measure remains unclear, it has been speculated that the recollective component may be driven by relative distinctiveness, whereas the familiarity-based component may instead be driven by differences in how the items are encoded; for example, it has been suggested that differential attentional engagement might account for the effect on familiarity (Fawcett, 2013; Fawcett & Ozubko, 2016; Ozubko et al., 2012). However, were this true, one might have expected our manipulation of alternating case to have interacted with the purported attentional component, as we described above. The fact that it did not may be viewed as questioning the role of attention in this paradigm. Importantly, the role played by attention in this paradigm is mixed – with studies such as ours (and those manipulating generation) demonstrating that forcing participants to deeply encode all items has little impact on the production effect, but other studies demonstrating an impaired production effect in populations with unmedicated attentional impairments (Mama & Icht, 2019) or when attention is drawn away via fluctuating-energetic noise (Mama et al., 2018). Furthermore, past studies have likewise shown that differences in effort between conditions at study are sometimes predictive of the behavioral production effect at test (Willoughby, 2020).

Despite observing varying degrees of evidence *against* a Production × Font interaction in each of our analyses, it is nonetheless worth considering whether our studies were adequately powered to detect such an interaction were it present. To determine the minimum effect size we could detect reliably, power simulations were undertaken using the *Superpower* package in *R* (Lakens & Caldwell, 2021) to audition a variety of plausible effects. We started by assuming overall mean performance like that observed for our analysis of hits in Experiment 1, with main effects of production and font, but no interaction. We then modified the means to reflect a reduction of the production effect for the alternating condition in steps of .01. As a reminder, our prediction was that the production effect would diminish – or even disappear – in the alternating case font condition. Power and the implied effect size associated with the interaction were calculated at each step. In short, we were roughly adequately powered in each study to detect a non-crossover interaction whereby the production effect was entirely eliminated within the alternating condition (aloud–silent = .00) while preserved in the lowercase condition (aloud–silent ∼ .12) for sample sizes matched to those in Experiment 1 (*n* = 34, power = 76%) and Experiment 2 (*n* = 43, power = 86%). However, this corresponds to a large effect (η_*p*_^2^ = .186). It is therefore possible that a more nuanced interaction between production and the mental effort associated with reading disfluent fonts was present but missed. Although possible, our findings are nonetheless buttressed by evidence, as quantified via Bayes factors suggesting that current findings are far more likely to suggest the absence of an interaction.

The present study examined how effortful reading might influence the relative benefit of production in one particular set of experimental conditions: when both the font type and production are manipulated within-subjects. It is important to note that although the production effect is observed between-subjects, it is smaller in magnitude (Fawcett, 2013; Fawcett et al., 2023) and shows the benefit in familiarity only, not in recollection (Fawcett & Ozubko, 2016), in contrast to the within-subjects production effect. Thus, it is possible that a different pattern of results would be observed if fluent and disfluent fonts were studied in pure lists read aloud or silently. Recently, Bodner et al. (2020) found that pure list production had little influence on source memory (while showing a production effect on item recognition), except in their final experiment in which participants had a sufficiently distinctive encoding task that supported recollection, which in turn supported source memory judgments. Speculatively, then, as we had observed increased recollection for words studied in alternating case font, we might predict similar findings if production were manipulated between-subjects: improved recognition for the group that read words aloud and a similar benefit for recognition of alternating case font words in both groups. One can also consider what might occur with the opposite design, with production manipulated in a mixed list, but with the font condition manipulated between-subjects. A production effect in the lowercase font group would be expected, and we might expect an overall memory benefit for the alternating case font group, given the slower reading times and increased recollection we observed. Whether an interaction would be observed in this case would be dependent on the degree to which the alternating case font encouraged encoding of orthographic features in the absence of lowercase font words in the same study context. These issues would be important to consider examining in future studies.

In conclusion, the present study replicated a typical mixed-list production task using consistent or alternating case at study to evaluate the role of differential encoding effort in the production effect. Although we observed a typical production effect, as well as improved memory for items presented in alternating case (in hits or recollection in Experiments 1 and 2, respectively), we failed to observe a significant interaction. This provides further evidence against the notion that the production effect is driven by a failure to attend to silent items, although further evidence is necessary to resolve disparate findings with the literature.

## Footnotes

^1^
Although several authors have expressed a preference for *d*′ within the production literature (e.g., Fawcett et al., 2012; Fawcett et al., 2023; Forrin et al., 2016), we have chosen to focus our analyses on hits because the aloud and silent items share a common false alarm rate and false alarms between font conditions were similar. Even so, comparable analyses conducted using *d’* produce equivalent results, with the sole exception that evidence favoring the Null was again slightly less convincing for the interaction, *F*(1, 33) = 2.80, *MSE* = 0.098 *p* = .104, η_*g*_^2^ = .004, *BF*_01_ = 2.09. Analysis of response bias was not undertaken owing to the common false alarm rate between our critical conditions. We thank an anonymous reviewer for suggesting this analysis.

^2^
Because the study was conducted online, it is unclear whether participant self-reports about complying with experiment instructions are in fact valid. As described above, the same words appeared in the same conditions for all participants, so there is potential for unanalyzed item effects to be influencing the results. We have also assumed that the alternating case font makes reading more effortful but have no independent measure of whether this is actually true.

^3^
The fact that each participant had only a single false alarm rate made conducting an analysis of *d’* unlikely to produce different results compared to the analysis of hits alone; therefore, these models were not undertaken. Similarly, analyses conducted excluding study phase trials for which bad responses had been made did not change any conclusions and had little impact on evidence for or against our effects.

^4^
This was pointed out by an anonymous reviewer; the partial overlap for this condition was indeed overlooked in the experimental planning stage.

## Notes

**Open Science:** To the best of my ability and knowledge, I have provided all original materials and clear references to all other materials via a stable online repository. All stimuli, data, and analyses reported in this article are available at https://doi.org/10.5281/zenodo.11246056 (jmfawcet, 2024).

## Appendix

**Table A1:** Lexical factors of word lists used in each condition in Experiment 1

Condition	Frequency	Familiarity	Imagability	Number of letters
Aloud				
Lowercase	257 (52.8)	570 (7.10)	447 (24.3)	5.8 (0.4)
Alternating	258 (47.0)	561 (9.06)	469 (29.7)	5.9 (0.3)
Silent				
Lowercase	354 (55.1)	578 (5.68)	429 (21.0)	5.2 (0.3)
Alternating	263 (50.4)	572 (7.48)	451 (28.3)	5.4 (0.3)
New				
Lowercase	279 (34.5)	584 (4.23)	484 (17.6)	5.3 (0.2)
Alternating	244 (47.6)	571 (5.56)	497 (15.6)	5.8 (0.2)
Mean	272 (19.4)	574 (2.57)	470 (8.83)	5.56 (0.114)
*Note*. Standard error of the mean is displayed within parentheses below the relevant mean. Each lexical factor was compared across the six lists using four separate 2 (font: lowercase vs. alternating case) × 3 (condition: aloud vs. silent vs. new) ANOVAs. The only analysis that showed a significant effect was the analysis on imagability (all other *p*s ≥ .085), which showed a significant main effect of condition, *F*(2,154) = 3.08, *p* = .049. Post hoc comparisons with Tukey correction showed that new words were marginally (*p* = .051) higher in imagability than silent words, but no other individual comparisons approached significance.				
